# An Aqueous Extract of a *Bifidobacterium* Species Induces Apoptosis and Inhibits Invasiveness of Non-Small Cell Lung Cancer Cells

**DOI:** 10.4014/jmb.1912.12054

**Published:** 2020-04-02

**Authors:** Joungjwa Ahn, Hyesung Kim, Kyung Mi Yang

**Affiliations:** 1 Department of Food Science and Technology, Jungwon University, Goesan-gun 28024, Republic of Korea; 2 Institute of Biomedical Science, Apple Tree Dental Hospital, Goyang 10387, Republic of Korea

**Keywords:** *Bifidobacterium*, probiotics, NSCLC cell, apoptosis, cancer invasion

## Abstract

Chemotherapy regimens for non–small cell lung cancer (NSCLC) have various adverse effects on the human body. For this reason, probiotics have received attention regarding their potential value as a safe and natural complementary strategy for cancer prevention. This study analyzed the anticancer effects of aqueous extracts of probiotic bacteria *Bifidobacterium bifidum* (BB), *Bifidobacterium longum* (BL), *Bifidobacterium lactis* (BLA), *Bifidobacterium infantis* 1 (BI1), and *Bifidobacterium infantis* 2 (BI2) on NSCLC cell lines. When the aqueous extracts of probiotic *Bifidobacterium* species were applied to the NSCLC cell lines A549, H1299, and HCC827, cell death increased considerably; in particular, the aqueous extracts from BB and BLA markedly reduced cell proliferation. p38 phosphorylation induced by BB aqueous extract increased the expression of cleaved caspase 3 and cleaved poly (ADP-ribose) polymerase (PARP), consequently inducing the apoptosis of A549 and H1299 cells. When the p38 inhibitor SB203580 was applied, phosphorylation of p38 decreased, and the expression of cleaved caspase 3 and cleaved PARP was also inhibited, resulting in a reduction of cell death. In addition, BB aqueous extracts reduced the secretion of MMP-9, leading to inhibition of cancer cell invasion. By contrast, after transfection of short hairpin RNA shMMP-9 (for a knockdown of MMP-9) into cancer cells, BB aqueous extracts treatment failed to suppress the cancer cell invasiveness. According to our results about their anticancer effects on NSCLC, probiotics consisting of *Bifidobacterium* species may be useful as adjunctive anticancer treatment in the future.

## Introduction

Lung cancer is the most common cause of cancer-related deaths worldwide, and non–small cell lung cancer (NSCLC) accounts for more than 85% of all lung cancer cases. Due to acquired drug resistance of NSCLC cells, surgical and chemotherapeutic treatments are unsatisfactory and may result in adverse effects [1-3]. Thus, it is necessary to find alternative therapeutic agents for lung cancer. Consequently, therapeutics derived from natural products, including a wide range of food ingredients and natural health foods, are in high demand and have gained increasing attention due to their intrinsic advantages [3, 4]. Among natural health foods that attracted much attention recently, probiotics, *i.e.*, nonpathogenic microorganisms living in the intestinal tract, offer various benefits to the host. Several existing in vivo and molecular studies have revealed antitumor effects of probiotic bacteria [5]. Studies on animal models and (human) clinical trials also showed positive effects of probiotics. Probiotics were effective in the treatment of acute diarrhea in clinical trials, and studies on inflammatory bowel disease also offer promising preliminary data [6, 7]. Besides, there are several studies showing inhibitory effects on cancer cell growth [8], and the proapoptotic potential [9] of microbial fermentation extracts.

Probiotics are living microorganisms that benefit the host by improving nutritional and microbial balance in the intestinal tract. Over the past few years, diverse anticancer properties of probiotics have been highlighted including protection of DNA from oxidative damage, immunomodulation, and a positive influence on carcinogenesis. Lactic acid bacteria (LAB), which have typical probiotic properties, can prevent intestinal infection by suppressing the invasion of human intestinal pathogens [10]. Several researchers have studied the antitumor effects of LAB [11, 12]. Recently, a growing number of studies revealed various effects of probiotics and highlighted their numerous health benefits. Different microbial species belonging to the genus *Lactobacillus* have been particularly interesting for the development of probiotics. LAB are also known as food additives present in various food products, such as dairy and meat products. Moreover, several studies have revealed the action of *Lactobacillus* and other LAB against carcinogenesis. Some species that have been found to be anticancer agents include *Lactobacillus rhamnosus*, *L. casei*, *L. bulgaricus*, and *L. acidophilus* [13]. *Bifidobacteria* are members of the normal microbiota of the human intestine. *Bifidobacterium infantis* has been shown to specifically target anaerobic tumor cells. Therefore, it is a good tumor-targeting gene therapy system, reported to effectively inhibit rat bladder tumor growth [14].

Despite increasing literature data on the promising effects of *Bifidobacterium* species against carcinogenesis, most of such studies have focused only on colon carcinoma cells. Little is known about the effects of *Bifidobacterium* species on NSCLC. Because it has been proven that the lungs host a variety of microbial communities, it is necessary to first determine the properties of every microorganism present there. One study implementing a probiotic therapy reported increased expression of genes inhibiting lung tumor growth along with an improved survival rate [15]. Because orally administered probiotics regulate an immune response in the respiratory system, the mechanisms of action in terms of prevention and treatment of respiratory diseases were also investigated [16]. Preclinical and clinical studies on the useful inhibitory effects of probiotics on solid tumor invasion in vitro will provide helpful evidence and help to determine the usefulness of administration of *Bifidobacterium* species to humans. Furthermore, it is necessary to demonstrate their positive effects in the lab to use them as a valid natural strategy to prevent cancer in the clinic. To validate this approach, the present study investigated the mechanism by which aqueous extracts of probiotic *Bifidobacterium* species exert anticancer activity on NSCLC cells.

## Materials and Methods

### Bacterial Strains and Extract Preparation 

The strains, *Bifidobacterium bifidum* 3440 (BB), *Bifidobacterium longum* 3128(BL), *Bifidobacterium lactis* 5854(BLA), *Bifidobacterium infantis* 3249 (BI1), and *Bifidobacterium infantis* 5934 (BI2) (Korean Collection for Type Culture, Korea), were incubated under anaerobic conditions (Bactron Anaerobic Chamber, Sheldon Manufacturing Inc., USA) at 37°C. Cell suspensions were washed twice via centrifugation and were resuspended in cold PBS. Cells were sonicated with an Ultrasonic Processor (Hielscher Ultrasonics GmbH., Germany) and then centrifuged at 12,000 ×*g* for 20 min. Supernatants were stored at -70°C until further analysis.

### Human Cancer Cell Culture

Human NSCLC cell lines A549, H1299, and HCC827 were purchased from the ATCC (American Type Culture Collection., USA). All the cells were cultured at 37°C and 5% CO_2_ in the RPMI medium supplemented with 10% of fetal bovine serum (FBS; HyClone Laboratories, Inc., USA) and with 10,000 U/ml solution of penicillin/streptomycin (Sigma–Aldrich., USA).

### WST-8 Assay

Cell viability was evaluated by a CCK-8 cell proliferation assay using the WST-8 assay kit (Dojindo Laboratory., Japan) as described previously [17]. A549 and H1299 cells in 96-well plates were cultured up to a density of 5 × 10^3^ cells/well. PBS served as a vehicle in a control group of cells. Following treatment with total aqueous extract of *Bifidobacterium* species for 24 h, the WST-8 colorimetric water-soluble tetrazolium dye was added into each well and incubated for 30 min. Absorbance was measured at 450 nm on an automated microplate reader (BioTeck Instruments., USA). All the experiments were conducted in triplicate.

### Western Blotting

Immunoblotting was performed to detect apoptosis–associated molecules. After BB treatment, the cells were lysed with RIPA buffer (Pierce, USA), and protein concentration was quantified by means of the Bradford protein assay (Bio-Rad, USA). A total cell lysate was prepared as described elsewhere [18]. Protein samples were resolved by SDS-PAGE and transferred onto a PVDF membrane (Bio-Rad). The membranes were probed with antibodies against cleaved caspase 3, cleaved PARP, p38, phosphorylated p38, Matrix Metalloproteinase-9(MMP-9) and β-actin (Cell Signaling Technology, USA). Signals were detected with secondary antibodies and Pierce ECL-Plus chemiluminescence (Thermo Scientific, USA).

### Propidium Iodide (PI) and Annexin V Staining 

DNA content was measured by PI staining of small DNA fragments followed by flow cytometry. Apoptotic cells that have lost DNA take up less stain and appear on the left side of the G1 peak in the histogram. Briefly, A549 cell groups were each seeded in wells of a 6-well plate and treated with BB and BLA extracts. All the cells were harvested and incubated at 4°C overnight, in the dark, with 750 μl of a hypotonic buffer (50 μg/ml PI in 0.1 %sodium citrate with 0.1% Triton X-100). Next, flow cytometry was conducted on a FACScan flow cytometer (Becton Dickinson., USA). A total of 10,000 events were acquired during the FACS analysis. Double staining and analysis of annexin V-FITC and PI reagents were performed according to manufacturer’s instructions

### An Enzyme-Linked Immunosorbent Assay (ELISA) 

The amounts of pro-MMP-9 released into the medium were measured by means of commercially available ELISA kits (R&D systems Inc., USA) according to the manufacturer’s instructions.

### Invasion Assay

Cell invasion was measured in 24-well chamber plates with polycarbonate membrane filter inserts of 8 μm pore size (Millipore Chemicon, USA). Cell invasion was determined using a Basement Membrane Matrix precoated with Matrigel (BD Biosciences, USA). A complete medium containing 10% of FBS served as a chemoattractant in the bottom chamber, and 2 × 10^5^ cells/ml were incubated with BB aqueous extract for 48 h. Invading cells were stained with 1% Sulforhodamine B (Sigma–Aldrich, USA) for 10 min, dried, and photographed. The bound dye was eluted with 10 mM Tris-HCl (pH 7.0) and quantified. The cell invasion ability was determined by measuring absorbance at 510 nm on the microplate reader (Thermo Labsystems, USA).

### MMP-9 Silencing

Predesigned small interfering RNA (siRNA) against MMP-9 was procured from Ambion Life Technologies. The sense siRNA sequence was 5’-GACCUGGGCAGAUUCCAAATT-3’. The antisense siRNA was 5’-UUUGGA AUCUGCCCAGGUCTG-3’. Transfection procedures were performed by means of the Mirus TransIT-X2 reagent (Mirus Corp., France) as per manufacturer’s instructions.

### Statistical Analysis

Data are shown as the mean ± SD and represent the mean of at least three separate experiments performed in triplicate. Differences between groups were determined by Student’s *t* test. Data are expressed as mean ± SD. Results with *p* < 0.01 were considered significant.

## Results

### The Aqueous Total Extract of the *Bifidobacterium* Species Increased the Cell Death and Inhibited Proliferation of NSCLC Cells

**Fig. 1:**
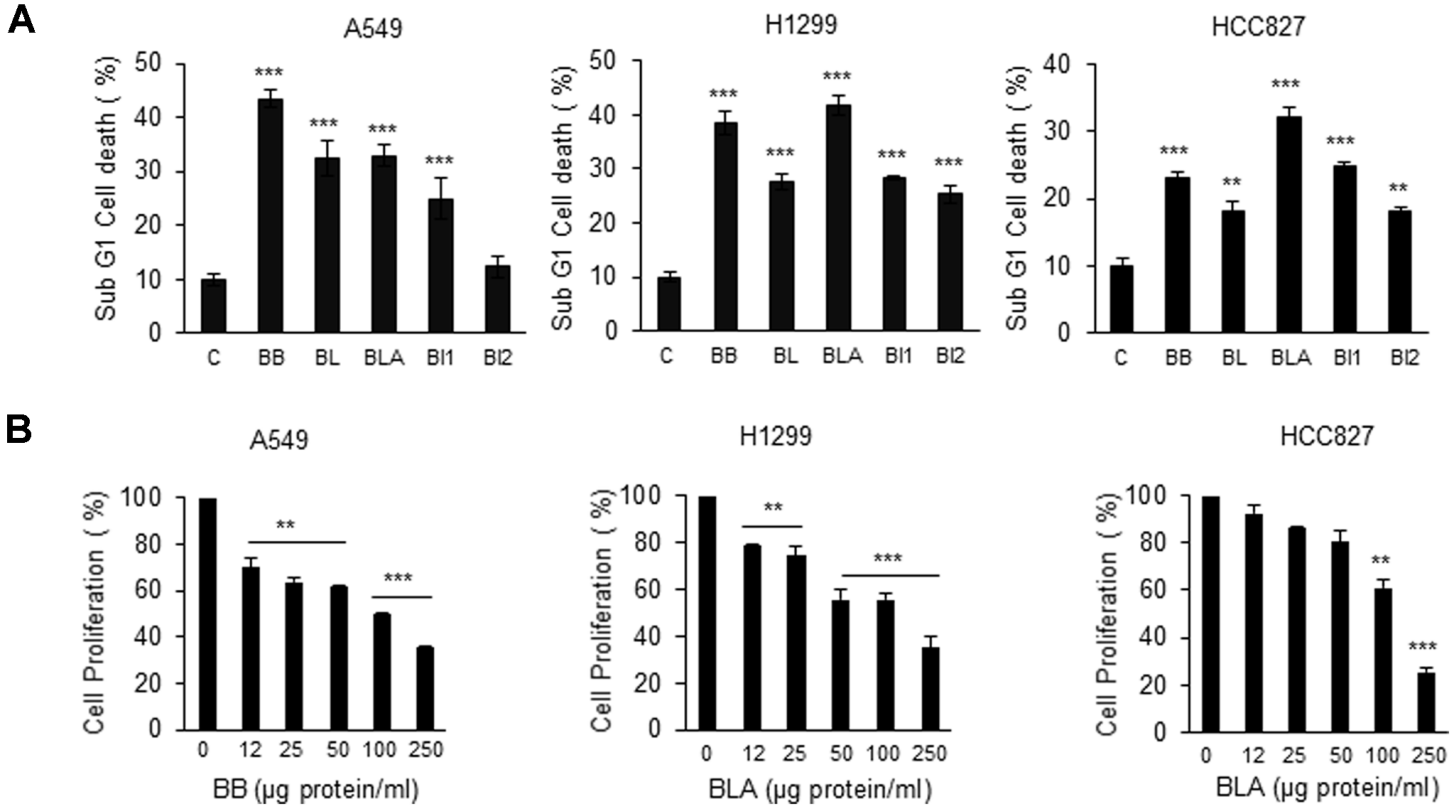
The total aqueous extract of *Bifidobacterium* species induced cell death and inhibited proliferation of NSCLC cells. (**A**) A549, H1299 and HCC827 (6 × 10^5^) strains were seeded in a 6-well plate. *Bifidobacterium bifidum* (BB), *Bifidobacterium longum* (BL), *Bifidobacterium lactis* (BLA), *Bifidobacterium infantis* 1 (BI1), and *Bifidobacterium infantis* 2 (BI2) were applied as aqueous extracts (150 μg protein/ml) and incubated with cells for 24 h. PBS served as a vehicle. The WST- 8 assay was performed to assess cell death. Values are expressed as the mean ± SD (**p < 0.01 and ***p < 0.001, as compared with the PBS treated Control). (**B**) A549, H1299 and HCC827 cells (6 × 10^5^) were seeded in a 6-well plate. BB or BLA aqueous extracts were added at 0, 12, 25, 50, 100, and 250 μg protein/ml on the following day, for 12 h incubation. WST-8 assay was performed to test cell proliferation. Values are expressed as the mean ± SD (**p < 0.01 and ***p < 0.001 as compared with the PBS treated Control).

In previous studies, we observed that cancer cell growth was considerably inhibited when dextrose served as a carbon source (data not shown). Accordingly, experiment was conducted to investigate the effect of aqueous extracts of *Bifidobacterium* species on the growth of various NSCLC cell lines. Aqueous extracts of BB, BL, BLA, BI1, and BI2 were cultivated in the culture medium supplemented with dextrose. The aqueous extracts were applied to A549, H1299, and HCC827 cells at 150 μg protein/ml. There was no death of cancer cells when nothing was treated as a cell only, and cancer cells death about 10% when PBS was treated as a control. Except for incubation of BI2 with A549 cells, the incubation with aqueous extracts noticeably increased cancer cell death as shown in Fig. 1A. In particular, when BB and BLA aqueous extracts were applied, proliferation of A549, H1299, and HCC827 cells considerably diminished in agreement with the increased cell death (Fig. 1B).

### Aqueous Extracts of the *Bifidobacterium* Species Induced Apoptosis of NSCLC Cells and Increased p38 Activity

**Fig. 2:**
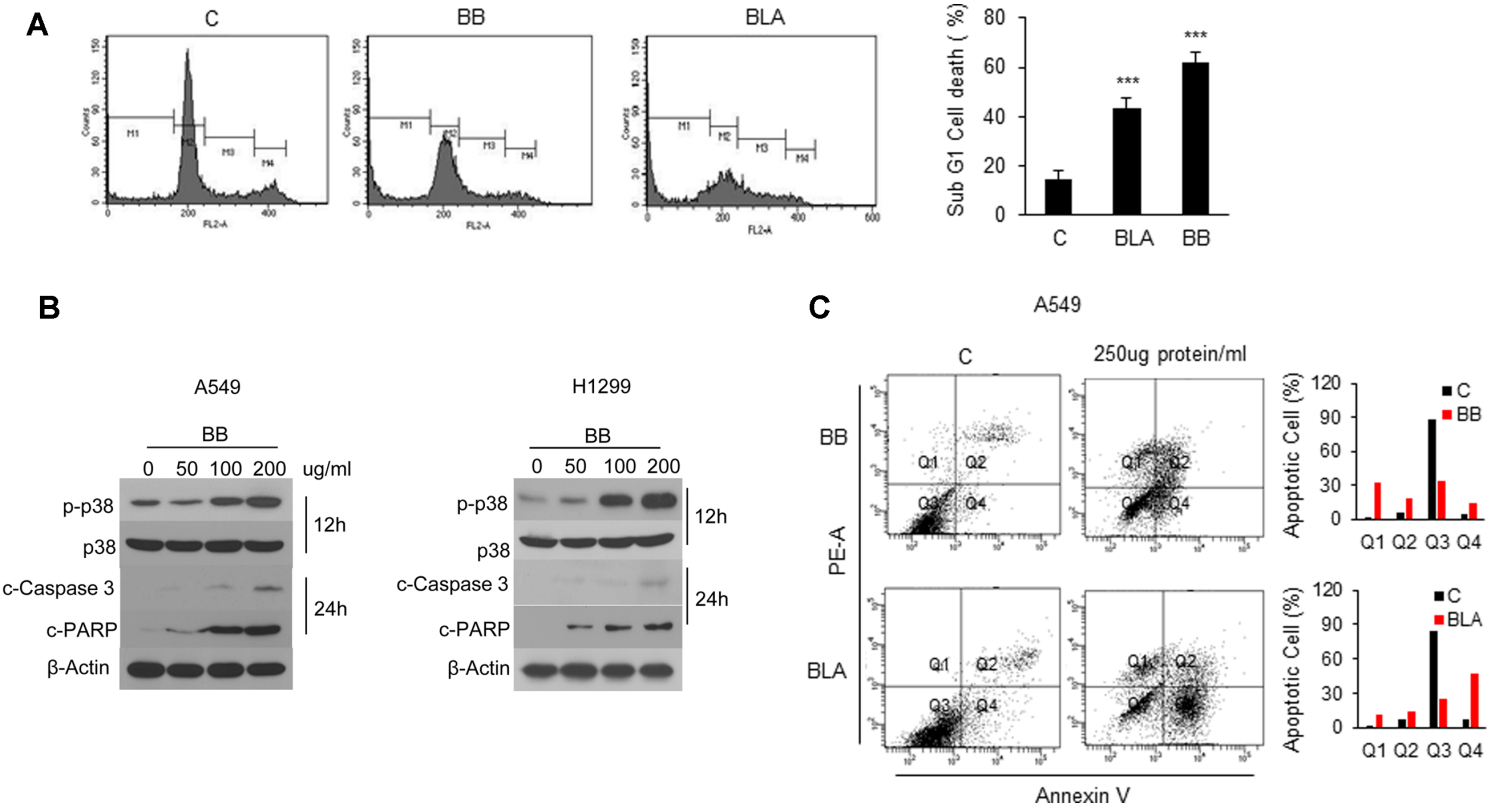
Apoptosis induced by *Bifidobacterium* aqueous extracts is associated with activation of p38 in NSCLC cells. (**A**) A549 cells (6 × 10^5^) were challenged with 150 μg protein/ml aqueous extracts of BB or BLA for 24 h. Propidium iodide (PI)-stained cells were analyzed by FACS. Data on apoptotic cells are presented as bar graphs. Values are expressed as the mean ± SD (***p < 0.001 as compared with the PBS treated Control). (**B**) A549 and H1299 cells (1 × 10^6^) were challenged with 50, 100, or 200 μg protein/ml or PBS control for the indicated periods. The levels of phospho- (p-)p38, p38, cleaved caspase 3, cleaved PARP, and β-actin were estimated by immunoblot analysis with specific antibodies. (**C**) A549 cells (5 × 10^5^) were challenged with 250 μg protein/ml aqueous extracts of BB or BLA for 30 h. Propidium iodide (PI)/Annexin V-stained cells were analyzed by FACS. Representative figures showing population of early apoptotic(Q4) and late apoptotic(Q2) cells.

DNA content degradation profiles were examined by PI staining to test whether the effects observed in Fig. 1 involve DNA fragmentation, which means apoptosis of NSCLC cells. As presented in Fig. 2A, BB and BLA aqueous extracts were applied to A549 cells, and then sub-G1 phase, DNA-containing population considerably increased when compared to the negative control without the bacterial extract. Quantification of the cell population supported these results. Because the apoptosis-inducing effect was observed, next, BB aqueous extracts was applied to A549 and H1299 cells at 0, 50, 100, or 200 μg protein/ml; and molecular changes in factors associated with apoptosis were analyzed by western blot analysis. p38 phosphorylation considerably increased as depicted in Fig. 2B, and cleaved caspase 3 and cleaved PARP became detectable in a time-dependent manner. PI /Annexin V analysis also showed that apoptosis increased with BB and BLA aqueous extracts at 250 μg protein/ml for 30 h. These results indicated that aqueous extracts of *Bifidobacterium* species induced apoptosis of NSCLC cells in a p38-activity-dependent manner.

### p38 Activity Is Indispensable for the Ability of *Bifidobacterium* Aqueous Extracts to Induce Apoptosis

**Fig. 3:**
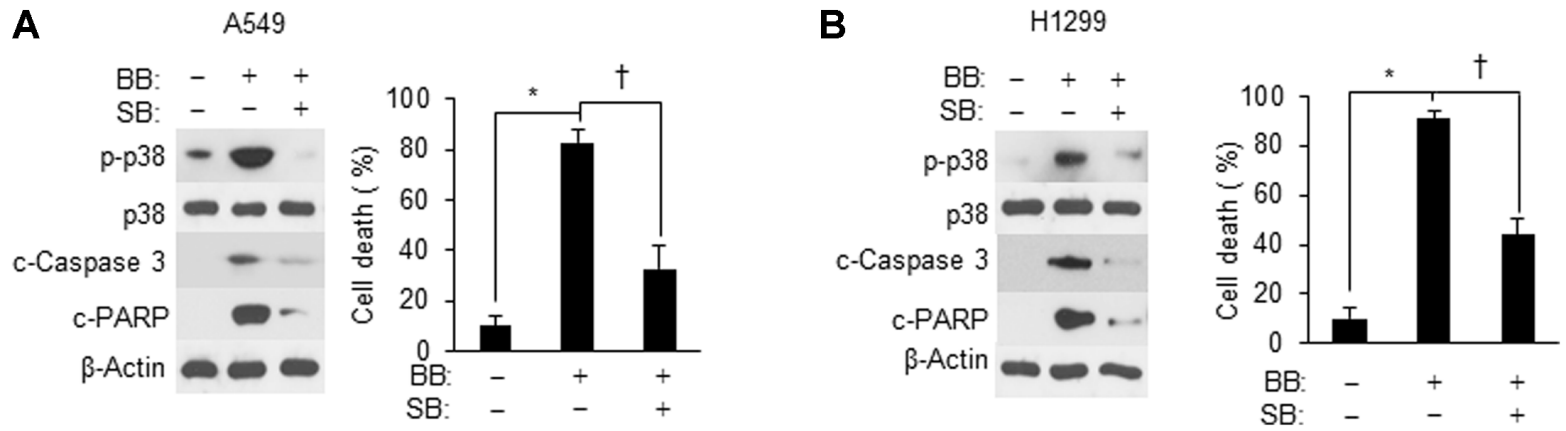
p38 activation is required for BB aqueous extracts -induced NSCLC cell apoptosis. (**A** and **B**) Cells (1 × 10^6^) were pretreated with SB203580 (20 μM), followed by BB aqueous extracts (100 μg protein/ml) treatment for 24 h. Protein samples were subjected to western blot analysis with antibodies against p-p38, p38, cleaved caspase 3, cleaved PARP, and β-actin. Cell death data quantified by the WST-8 assay are presented as bar graphs. Values are expressed as the mean ± SD (*p < 0.05 as compared with the PBS treated group; ^†^*p* < 0.05 as compared with the BB aqueous extracts -treated group).

To clearly demonstrate that *Bifidobacterium* aqueous extracts induced apoptosis in NSCLC cells in a p38-activity-dependent manner, SB203580 was used as an inhibitor to suppress p38 activity, followed by BB aqueous extracts treatment. As illustrated in Fig. 3, p38 phosphorylation, which increased after BB aqueous extracts application to A549 and H1299 cells, significantly decreased in the presence of inhibitor; and the cell death rate also dramatically diminished. These data suggested that p38 activity is necessary for BB aqueous extracts-induced apoptosis.

**Fig. 4:**
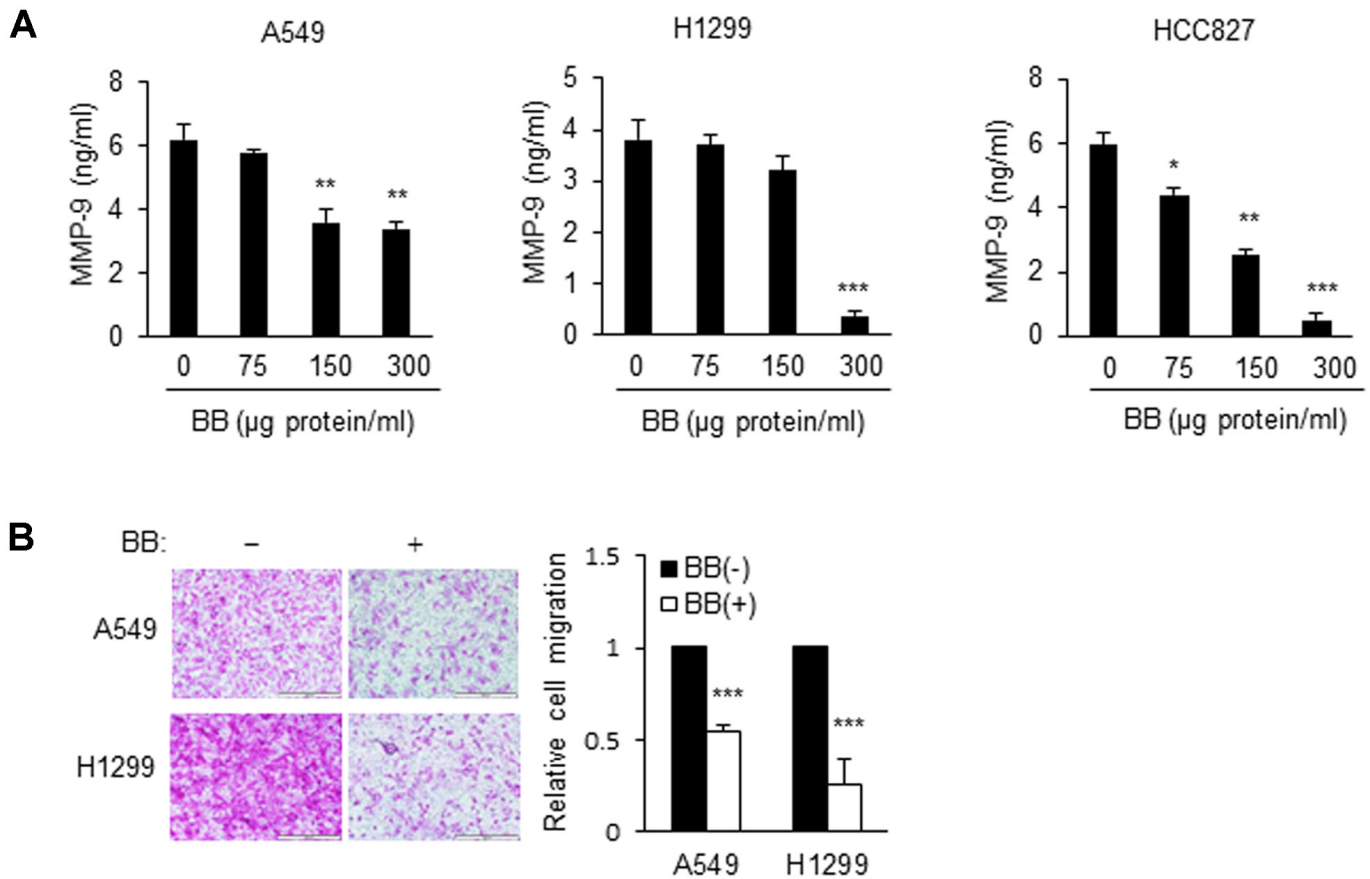
BB aqueous extracts decreases the level of MMP-9 in NSCLC cells. (**A**) Cells (2 × 10^6^) were treated with BB aqueous extracts at indicated concentrations (0, 75, 150, or 300 μg protein/ml) for 24 h, and the levels of MMP-9 were determined by an ELISA. Values are expressed as the mean ± SD (*p < 0.05, **p < 0.01, and ***p < 0.001 as compared with the PBS treated group). (**B**) BB aqueous extracts inhibited the invasiveness of A549 and H1299 cells. Cells (2 × 10^5^) were exposed to 100 μg protein/ml BB aqueous extracts for the indicated periods. Invasion was assessed by the chemotactic Transwell assay. Original magnification, 200×. Values are expressed as the mean ± SD (***p < 0.001 as compared with the PBS treated group).

*Bifidobacterium* Aqueous Extracts Inhibit Cancer Cell Invasion by Regulating MMP-9 in NSCLC Cells Matrix metalloproteinase 9 (MMP-9) is known to play an important role in cancer cell invasion and metastasis. To test whether suppression of NSCLC invasion by BB aqueous extracts is mediated by MMP-9, the MMP-9 levels in culture supernatants of BB aqueous extracts-treated A549, H1299, and HCC827 cells were estimated. As shown in Fig. 4A, the MMP-9 level decreased significantly when BB aqueous extracts was applied. In contrast, the MMP-9 level showed no reduction in BB aqueous extracts-treated NSCLC cell strains in the RT-PCR assay (Fig. 4B). Thereafter, the effect of extracts on invasion was analyzed in Matrigel-coated Transwell plates. As presented in Fig. 4B, the level of invasion diminished after incubation with BB aqueous extracts. These results indicated that the invasion of highly metastatic NSCLC cells was suppressed by BB aqueous extracts. Conversely, the secretion of GROa, IL-6, IL-8, and CCL5 cytokine increased when BB aqueous extracts was incubated with A549 cells. Therefore, the cytokine profile did not sufficiently support the invasion-suppressive effect (data not shown).

### MMP-9 Knockdown NSCLC Cells Rescue to Invade

**Fig. 5:**
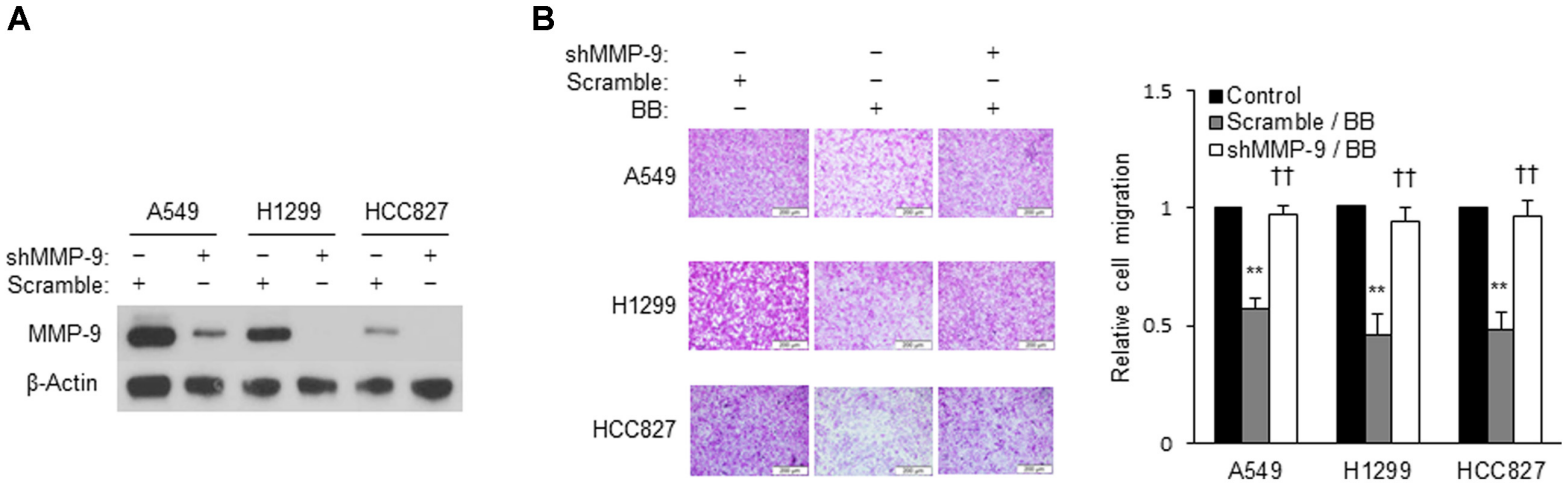
Inhibition of MMP-9 expression failed to reduce invasion by BB aqueous extracts treatment in NSCLC cells. (**A**) Western blotting for MMP-9 expression in MMP-9 knockdown cells. MMP-9 knockdown was performed with the shRNA system. Cells (5 × 10^5^) were transfected with the shRNA using viafect reagent according to the manufacturer's instructions. (**B**) Transfected cells invasion was measured using matrigel coated in 24-transwell chamber plates. BB aqueous extracts containing in the upper chamber and cells (2 × 10^5^) were incubated for 48 h. Invaded cells were visualized by SRB staining and eluted by 10 mM Tris-HCl buffer. Original magnification (200×). Values are expressed as the mean ± SD (**p < 0.01 as compared with the Scramble; ^††^*p* < 0.01 as compared with the BB aqueous extracts -treated group).

As depicted in Fig. 4, incubation with BB aqueous extracts resulted in inhibition of MMP-9 secretion by NSCLC cells along with suppression of invasion. Therefore, we tested whether MMP-9 plays an important role in BB aqueous extracts -induced inhibition of invasion. To this end, MMP-9 in NSCLC cells was silenced first. As shown in Fig. 5A, lung cancer cell lines with successfully downregulated MMP-9 expression were selected by western blotting. BB aqueous extracts was incubated with these MMP-9 knockdown cell strains. BB aqueous extracts reduced the invasion when it was applied to the cell line transfected with a scrambled short hairpin RNA (which does not downregulate MMP-9). By contrast, the cancer cell invasion was rescued by transfection with siMMP-9 (which caused a knockdown of MMP-9). These findings suggested that MMP-9 is very important for BB aqueous extracts treatment to reduce the invasiveness of NSCLC cells.

## Discussion

Interest in the benefits of probiotic *Bifidobacterium* species for human health continues to grow [19, 20]. *Bifidobacterium* species living in the large intestine help maintain human health, and the disappearance or reduction of the *Bifidobacterium* population signals a problematic state of health. Many studies suggest that oral administration of bifidobacteria may be effective at improving the intestinal environment, by alleviating intestinal and liver disorders, stimulating immune responses, preventing cancer, and delaying aging [21-23]. Microbiota distribution is not limited to the intestine; these beneficial bacteria are found in various tissues. Thus, several researchers lately shifted their attention to various other topics. The lungs, in particular, have been previously considered free of a microbiota in a good state of health, but it was recently discovered that a variety of microbial communities live in the lungs, in the lower part of the respiratory tract [24, 25]. Studies on the lung microbiota are rapidly increasing in number, and a growing number of important findings has been reported in relation to cancer [26].

For the first time, our study demonstrated the anti-NSCLC effects of total aqueous extracts of *Bifidobacterium* species, for example, induction of apoptosis and suppression of invasiveness of NSCLC cells. When *Bifidobacterium* extracts were incubated with NSCLC cell lines A549, H1299, and HCC827, p38 was activated in a concentration-dependent manner. The expression of apoptosis indicator molecules, cleaved caspase 3 and cleaved PARP, considerably increased at a later time point. These treatments induced apoptosis and suppressed cell proliferation, and these effects were attenuated by treatment with SB203580, a p38 inhibitor. These results indicated that p38 activation must be highly important for induction of apoptosis in NSCLC by bifidobacteria. Similarly, a previous study revealed that the supernatant of human-derived *Lactobacillus reuteri* promotes tumor necrosis factor (TNF)-induced apoptosis of human chronic myeloid leukemia cells through p38 activation [27].

There have been no reports on induction of apoptosis through p38 MAPK phosphorylation in NSCLC cells. In a study that investigated the anticancer effect of bifidobacteria extracts, a butanol extract of *Bifidobacterium adolescentis* isolated from healthy young Koreans exerted an antiproliferative action on colon cancer cells; for this effect, tumor necrosis factor-α (TNF-α) and nitric oxide (NO) were found to be important [28]. *Lactobacillus casei* extracts induce apoptosis of gastric cancer cells through NF-κB and mTOR-mediated signaling [29]. Therefore, the cell proliferation–regulatory and cell death–regulatory properties of probiotic bacteria may be highly useful as a cancer prevention strategy. In addition, *Lactobacillus* species effectively suppress malignant phenotypes of colorectal cancer cells [30]. The cell-free supernatants of *Lactobacillus* sp., *Lactobacillus casei*, and *Lactobacillus rhamnosus* GG reduce metalloproteinase 9 (MMP-9) activity and increase the expression of a tight junction protein, zona occludens 1 (ZO-1), thereby inhibiting the invasiveness of colorectal carcinoma cells [31].

Tumor invasion is a result of the direct expansion of cancer cells and their penetration of adjacent tissues. The proliferation of transformed cells and the gradual expansion of the tumor eventually destroy the barriers between tissues, facilitating the spread of the tumor to adjacent tissues. Local invasion is the first step that leads to the process of secondary-tumor formation or metastatic progression [32]. MMPs are zinc-dependent endopeptidases that can degrade all components of the extracellular matrix (ECM) [33]. In the present study, we observed that, among members of the MMP family, MMP-9 levels decreased in a concentration-dependent manner according to an ELISA assay, when *Bifidobacterium* extracts were incubated with the NSCLC cell lines. The aqueous total extracts of *Lactobacillus* species (*L. acidophilus* and *L. casei*) also effectively inhibit the malignant properties when applied to a colorectal tumor cell line: CaCo2 cells [30]. On the other hand, the cell-free supernatant (CFS) of *L. casei* and *L. rhamnosus* GG prevents the invasion of colon cancer cells, suggesting that the probiotic CFS probably contains antimetastatic bioactive substances involved in *in vitro* cell disintegration [31]. According to a recent study, *L. rhamnosus* and *L. crispatus* CFSs reduce the expression of matrix metalloproteinase 2 (MMP-2) and MMP-9 in HeLa cells and increase the expression of their inhibitors. *L. rhamnosus* has manifested a similar effect in HT-29 cells [34]. In addition, *L. acidophilus* and *L. rhamnosus* GG suppress the expression of MMP-9 through upregulation of metalloproteinase 1 (TIMP) inhibitor and downregulation of CD147 during phorbol 12-myristate 13-acetate–induced differentiation [35].

Judging by our results on NSCLC cells, the number of invading cancer cells decreased when *Bifidobacterium* extracts were applied. In contrast, the extracts failed to reduce this number when applied after transfection of shMMP-9 (which causes a knockdown of MMP-9). These findings indicate that BB aqueous extracts caused the cancer cell invasion through MMP-9 suppression. It is known that various cytokines contribute to the process of cancer cell metastasis[36]. Contrary to our expectation, however, the secretion of proinflammatory cytokines increased after the treatment with probiotics. Further research is urgently needed to validate this observation. Similarly, another study revealed that proinflammatory, rather than anti-inflammatory, cytokine secretion is induced when adult-type bifidobacteria are incubated with a murine macrophage‐like cell line, J774.1 [37]. Conversely, Medina *et al*. reported that *Bifidobacterium* strains can properly downregulate proinflammatory cytokines that were induced by large intestinal microbiota in coeliac patients in an in vitro experiment [38].

There are still unexplained mechanisms underlying the effects of probiotics on inflammatory responses; therefore, it is necessary to take a multidisciplinary approach to understand them. Because it has been shown that the lungs host a diverse microbiota, more studies are needed to shed light on the regulatory mechanisms to understand how each microorganism contributes to health benefits.

In conclusion, probiotic *Bifidobacterium* supplementation induced apoptosis in NSCLC cells and inhibited their invasiveness through MMP-9 downregulation. According to these findings, it is likely that *Bifidobacterium* species can enhance cancer treatment as symbiotic microorganisms and will serve as a safe complementary strategy for cancer prevention in the future.
